# Fabrication and Characterization of Novel Silk Fiber-Optic SERS Sensor with Uniform Assembly of Gold Nanoparticles

**DOI:** 10.3390/s22229012

**Published:** 2022-11-21

**Authors:** Taeyoung Kang, Yongjun Cho, Kyeong Min Yuk, Chan Yeong Yu, Seung Ho Choi, Kyung Min Byun

**Affiliations:** 1 Department of Electronics and Information Convergence Engineering, Kyung Hee University, Yongin 17104, Republic of Korea; 2 Department of Biomedical Engineering, Kyung Hee University, Yongin 17104, Republic of Korea; 3 Department of Biomedical Engineering, Yonsei University, Wonju 26493, Republic of Korea

**Keywords:** silk fibroin-based optical fiber, convective self-assembly, surface-enhanced Raman spectroscopy, gold nanoparticles

## Abstract

Biocompatible optical fibers and waveguides are gaining attention as promising platforms for implantable biophotonic devices. Recently, the distinct properties of silk fibroin were extensively explored because of its unique advantages, including flexibility, process compatibility, long-term biosafety, and controllable biodegradability for in vitro and in vivo biomedical applications. In this study, we developed a novel silk fiber for a sensitive optical sensor based on surface-enhanced Raman spectroscopy (SERS). In contrast to conventional plasmonic nanostructures, which employ expensive and time-consuming fabrication processes, gold nanoparticles were uniformly patterned on the top surface of the fiber employing a simple and cost-effective convective self-assembly technique. The fabricated silk fiber-optic SERS probe presented a good performance in terms of detection limit, sensitivity, and linearity. In particular, the uniform pattern of gold nanoparticles contributed to a highly linear sensing feature compared to the commercial multi-mode fiber sample with an irregular and aggregated distribution of gold nanoparticles. Through further optimization, silk-based fiber-optic probes can function as useful tools for highly sensitive, cost-effective, and easily tailored biophotonic platforms, thereby offering new capabilities for future implantable SERS devices.

## 1. Introduction

Biophotonic components are essential for practical use in biomedical applications such as sensors, imaging, diagnostics and therapeutics [1,2,3,4,5]. Among them, optical fibers and waveguides are of specific interest because of their ability to manipulate and transport light in a controlled manner. In many biomedical areas, these functional elements must interface directly with living cells and tissues, requiring the materials to be biocompatible [6]. Especially for a biophotonic implantable device, it may also be demanded that the components be biodegradable. Hence, the use of biocompatible and biodegradable materials to guide light would open new opportunities for minimally invasive and flexible sensing platforms with the potential for biomedical utility as well as environmental compatibility [7,8].

Silk fibroin, a natural protein extracted from *Bombyx mori* cocoon, has been employed to serve as a biological supporting material in the various fields of neural prosthetics, tissue engineering and drug delivery because of its biocompatibility, biodegradability and lack of inflammatory response in vivo [9,10]. A soft and flexible silk fibroin enables intimate contact with the skin, tissues, or organs. Transparent and process-compatible silk fibroin facilitates its integration with functional materials, providing great convenience for micro/nanofabrication processes [11]. In addition, silk fibroin has a slow and controllable degradation rate and the ability to be fabricated into multiple forms of fibers, films and gels [12,13]. Consequently, it has emerged as an ideal biomaterial of choice for the design and integration of implantable biosensing platforms.

Surface-enhanced Raman spectroscopy (SERS) is a potential biosensing technique that allows for highly specific and sensitive molecular diagnostics. The substantial enhancement of Raman scattering for small molecules near electromagnetic hotspots of plasmonic nanostructures results in an extraordinary SERS sensitivity [14]. Since a weak Raman signal is often overwhelmed by the fluorescence background, many researchers have been forced to incorporate metallic nanostructures onto the substrate to amplify the Raman signal [15,16]. For next-generation SERS sensors, wearable or implantable platforms for personalized diagnosis are requisite to monitor important biomolecules inside the body. Such in vitro and in vivo SERS approaches should be supported using soft, flexible, biocompatible, and biodegradable substrates.

However, the existing SERS-active optical fiber structures are not directly suitable for implantable sensing applications due to the lack of biocompatibility and biodegradability [17]. For example, the tapered silica fibers decorated by metallic nanostructures were developed for high sensitivity through simple chemical corrosion or a mechanical method with mass production [18,19,20]. Nonetheless, the silica glass material is not biocompatible, and this hinders the practical realization of the implantable SERS sensors. In addition, conventional fiber optic sensors based on plasmonic nanostructures often require expensive equipment and time-consuming production processes [21,22]. Although alternative approaches, such as breath figure methodologies and nanosphere lithography, are cost-effective and efficient for regularly patterned nanostructures [23,24], these methods still demand professional operation and specialized equipment for the process steps, such as fiber polishing, spin coating, and plasma etching.

To the best of our knowledge, this study is the first to report a simple and economical fabrication of fiber-optic probes based on biological silk fibroin for a sensitive SERS sensor platform. The silk optical fiber comprises a core and a cladding obtained from the regenerated silk fibroin. The obtained silk fiber is soft, smooth, and flexible, which can be advantageous for biomedical devices. The implementation of plasmonic nanostructures on a fiber-top surface was achieved using a cost-effective nanofabrication method, that is, the convective self-assembly (CSA) of gold nanoparticles [25]. The constructed gold nanoparticles allowed strong electromagnetic field enhancement and good spatial uniformity, which is critical for sensitive and reliable SERS detection. Moreover, the use of the proposed silk-based fiber-optic probe as an effective SERS substrate was verified and validated through comparisons of various Raman scattering experimental results with those of commercial multi-mode optical fibers.

## 2. Materials and Methods

### 2.1. Materials

Distilled water (H_2_O), methanol (CH_3_OH), acetone (C_3_H_6_O), ethyl alcohol (C_2_H_5_OH), and hydrogen peroxide (H_2_O_2_) were purchased from DaeJung Chemicals and Metals Co., Ltd. (Siheung, Korea). Lithium bromide (LiBr), sodium carbonate (Na_2_CO_3_), hexafluoro-iso-propanol (HFIP), horseradish peroxidase (HRP), and 4-aminobenzenethiol (4-ABT, C_6_H_7_NS) were purchased from Sigma-Aldrich (St. Louis, MO, USA). Gold nanoparticles with an average diameter of 30 nm were purchased from Nanopartz (Loveland, CO, USA). *Bombyx mori* cocoons were supported by the Rural Development Administration (Wanju, Korea).

### 2.2. Fabrication of Silk Optical Fiber

**Figure 1:**
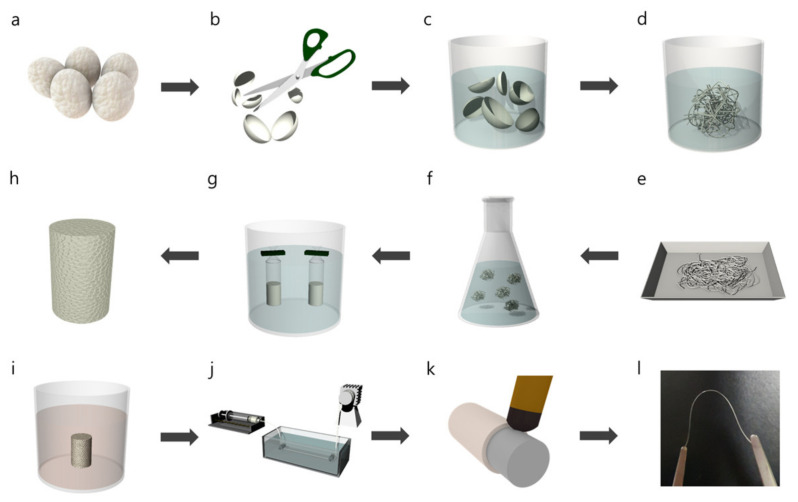
Fabrication process of silk optical fibers. (**a**) Preparing *Bombyx mori* cocoons. (**b**) Cutting. (**c**) Degumming. (**d**) Removing sericin. (**e**) Drying. (**f**) Dissolving in LiBr. (**g**) Dialysis. (**h**) Freeze-drying. (**i**) Dissolving in HFIP. (**j**) Wet spinning. (**k**) Cladding coating. (**l**) Photo of the silk optical fiber.

The fabrication procedure of a silk-based fiber-optic probe is demonstrated in Figure 1. Extraction of silk fibroin proceeded from *Bombyx mori* cocoon as the starting material (Figure 1a). The silk is composed of fibroin protein and glue-like sericin. As sericin is known to induce inflammatory responses in biomedical applications [10], it was removed through a degumming step. To effectively remove sericin, *Bombyx mori* cocoons were cut into pieces (Figure 1b) and boiled in 0.02 M sodium carbonate for 30 min (Figure 1c). Subsequently, the obtained silk fibroin bundles were rinsed in distilled water for 20 min to remove any residual chemicals (Figure 1d). After repeating the rinsing process twice, the fibroin bundle was squeezed and dried overnight at room temperature (Figure 1e). The fibroin bundle was then dissolved in 9.3 M LiBr solution at 60 °C for 4 h (Figure 1f). Thereafter, the fully dissolved solution was filtered using Miracloth to remove any artifacts. Further purification of the solution was achieved via dialysis (Figure 1g). The filtered solution was then injected into a dialysis membrane tubing (12–14 kDa MWCO, Fisher Scientific) and then dialyzed against distilled water at 5 °C for 2 days. The dialysis water was changed periodically during the 2-day span. The aqueous silk solution was then lyophilized for 2 days to obtain silk fibroin powder (Figure 1h). A 15 wt% solvent-based silk fibroin solution was prepared by dissolving the silk fibroin powder in HFIP at room temperature for 24 h (Figure 1i). The solution was spun into a methanol bath using a syringe pump at a speed of 30 mL/h and a 25-gauge needle tip (Figure 1j). Solidification and crystallization were promoted by leaving the filaments in a methanol coagulation bath for 12 h. The filaments were then swelled in a distilled water bath at 70 °C for 20 min to remove the residual methanol. After drying the filaments at room temperature, a silk fiber core was obtained without any shrinkage. Subsequently, silk fibroin hydrogel, a mixture of pure silk fibroin solution, HRP, and H_2_O_2_, was employed as the cladding material [26]. They were mixed on a hot plate at a temperature of 37 °C for 5 min, which slowly changed the solution to a gel form. Thereafter, the silk hydrogel was uniformly coated on the silk core during the intermediate phase between the liquid and solid phases (Figure 1k). Following the complete hardening of the coating at room temperature, the silk fiber was dipped into a 50% ethanol solution for 18 h and dried with nitrogen gas. Finally, soft and flexible silk optical fibers were successfully fabricated (Figure 1l).

### 2.3. Adsorption of Gold Nanoparticles Using CSA Method

The top surfaces of the silk and silica multi-mode optical fibers were cleaned via sonication in acetone and ethyl alcohol sequentially for 10 min and rinsed with distilled water and ethyl alcohol for 5 min, and then dried with nitrogen gas. Hydrophilic fiber-top surfaces were vertically immersed into gold nanoparticle suspension with a concentration of 5 optical density and at a temperature of 40 °C. Kapton tape was used as a protective layer to avoid the adsorption of gold nanoparticles onto the other sides of the optical fibers. We could observe a specific CSA feature of the meniscus tip at the interface between the substrate surface and gold nanoparticle suspension. Gold nanoparticles were deposited at the meniscus tip region via evaporation, which is associated with evaporation-induced upward convection [27,28]. Moreover, the attached gold nanoparticles were strongly bound to the fiber-top surfaces, and no destruction or damage to the pattern was found during or after the washing, cleaning, and binding processes.

### 2.4. SERS Experiments

In the SERS experiments, 4-ABT molecules, also known as para-aminothiophenol, were chosen as the Raman probe. 4-ABT can be specifically immobilized via thiol groups. 4-ABT was also used as a linker to tightly combine ligands with nanoparticles because of its special double-functional group characteristic, which is crucial to Raman immune detection [29]. After the SERS substrates were immersed into the 4-ABT solution for 15 min to induce chemical binding, the substrates were rinsed with ethyl alcohol and distilled water for 5 min to remove non-immobilized 4-ABT. The Raman spectra of 4-ABT were obtained at five different points in each given sample with an acquisition time of 50 s at 10× magnification. The experimental setup for the Raman spectra measurements comprised a microscope (BX43, Olympus, Tokyo, Japan), a continuous wave laser of λ = 785 nm (I0785MM0350MF, Innovative Photonic Solutions, Plainsboro Township, NJ, USA), a spectrometer (SR-303i-A, Andor Technology, Belfast, UK) and a low dark current deep-depletion CCD detector (iVac, Andor Technology, Belfast, UK).

## 3. Results and Discussion

**Figure 2:**
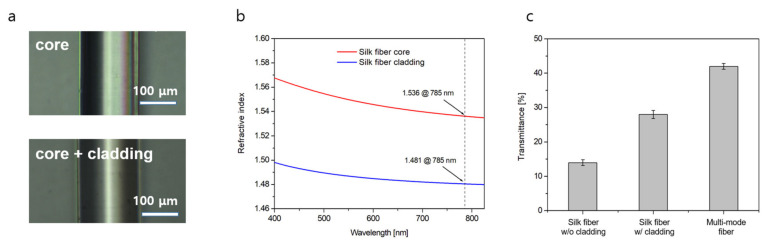
Characteristics of the silk fiber-optic probe. (**a**) The outer diameter of the silk core and cladding layers. (**b**) Refractive index measure for core and cladding structures made of silk fibroin proteins. The dotted line at λ = 785 nm indicates the condition for the SERS experiment. (**c**) Optical transmittance test results for silk fibers with and without a cladding layer and multi-mode fibers. The fiber length was fixed at 3 cm.

By processing the silk proteins, as shown in Figure 1, we fabricated a biological silk optical fiber, which was composed of a core of silk fibroin and a cladding of silk fibroin hydrogel. Figure 2a shows optical microscope images of the silk fiber-optic probe with and without a cladding layer. The average outer diameter of the bare silk fibroin core was measured to be 140 μm. Upon the application of a thin silk hydrogel coating, a core/cladding structure measuring 150 μm in diameter was obtained. For both the core and cladding materials, wavelength-dependent refractive index profiles were characterized by using a refractometer instrument (2010/M, Metricon Corporation, Pennington, NJ, USA), as presented in Figure 2b. The cladding layer was found to have a refractive index of 1481 at λ = 785 nm, which was slightly lower than that of the silk core (1536). A higher refractive index of the silk core is necessary to allow for light confinement inside the silk optical fiber. Notably, the refractive indices of the core and cladding exceeded that of water, indicating that the silk fiber fulfills the requirements for guidance within a water-based biological environment [6].

To measure the quality of light guiding, the optical transmission characteristics of silk and multi-mode optical fibers measuring 3 cm in length were tested using a He:Ne laser source (λ = 632.8 nm, 7.8 mW, Thorlabs, Newton, NJ, USA). As shown in Figure 2c, the optical transmittance values of the silk optical fibers were 28 ± 1.2 and 14 ± 0.9% for cases with and without a cladding layer, respectively. The introduction of the cladding layer improved the transmittance through the fibers by more than two times via the principle of total internal reflection [30]. For comparison, the average transmittance of silica multi-mode fibers (FG200LEP, Thorlabs, core/cladding diameter 220 μm) was 42 ± 0.9% under the same measurement conditions. Although the transmittance value of the silk optical fiber with a cladding layer was slightly reduced owing to the contrasts in the materials and geometric parameters of the core and cladding layers, the results were comparable to those observed for commercial products. A small standard deviation of less than 3% further indicates the quality of the silk optical fibers. Thus, the features of optical transmittance and its consistency for the fabricated silk-based fiber-optic probes were favorably evaluated, proving that they may find potential applications in a variety of optical devices.

**Figure 3:**
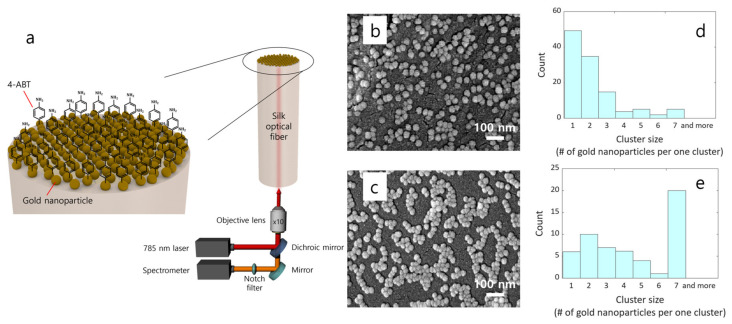
Silk-based fiber-optic SERS probe with gold nanoparticles (**a**). A schematic diagram of the SERS measurement setup and FE-SEM images of gold nanoparticles distributed on top of (**b**) a silk fiber and (**c**) a multi-mode fiber are presented. Histograms of the cluster size distribution were computed for (**d**) the silk and (**e**) the silica multi-mode fibers, respectively.

Figure 3a illustrates a schematic diagram of the fiber-optic probe and the SERS measurement setup. An excitation light of λ = 785 nm and an optical power of 20 mW were coupled through the fiber end. The SERS signals of 4-ABT molecules at the top surface were collected by the spectrometer with an acquisition time of 50 s after passing through the fiber waveguide, the objective lens of 10× magnification, mirrors and notch filter. The 3-axis motorized stage with a working distance of 5.5 mm was adjusted manually through a microscope to fix and focus the light into the fiber precisely.

CSA technique effectively constructed plasmonic gold nanoparticles on the fiber-top surface, which induced strong electromagnetic hotspots and thus enabled sensitive SERS detection of target molecules. A field-emission scanning electron microscope (FE-SEM) image in Figure 3b presents the morphology of gold nanoparticles for silk fibers. For comparison with the results by a typical fiber substrate, gold nanoparticles were assembled onto the silica multi-mode fiber using the same CSA protocol (Figure 3c). The SEM images indicate that gold nanoparticles were not highly aggregated on the top surface of the silk fiber. On the other hand, the surface of the multi-mode fiber exhibited a high aggregation of gold nanoparticles. From the histogram of the cluster size distribution in Figure 3d,e single gold nanoparticle with the largest number was dominant and highly aggregated gold nanoparticles were rarely found for the silk fiber. In contrast, a very large cluster including more than 7 gold nanoparticles showed the highest number in the case of the silica multi-mode fiber, implying that the high aggregation feature is associated with the non-uniform distribution of gold nanoparticles. As the immobilization features of the CSA technique are dependent on the degree of hydrophilicity of the substrate surface [28,31], the silk and silica multi-mode fiber cores induced the different distributions of gold nanoparticles on the top surface of individual fiber samples. Consequently, such a contrast in the distribution of gold nanoparticles may affect sensor characteristics, such as sensitivity and reliability.

**Figure 4:**
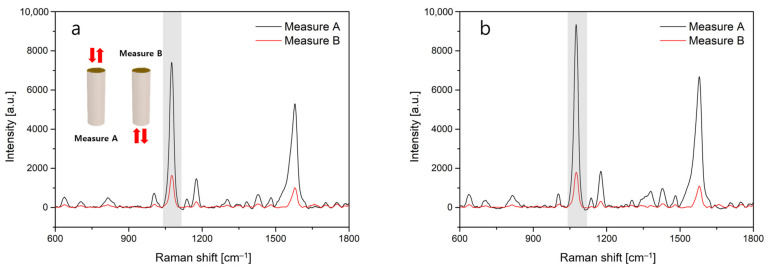
Measured SERS signals of 4-ABT molecules when laser light is incident to the fiber-top surface directly (Measure A) and when the light is coupled through the core/cladding structure (Measure B) for (**a**) a silk fiber and (**b**) a multi-mode fiber. The gray bar indicates the primary peak of 4-ABT molecules. The fiber length was fixed as 1 cm for both samples.

Subsequently, we measured the Raman scattering signals of 4-ABT molecules as the target analyte. After the fiber-optic SERS probes with a length of 1 cm were immersed into the 4-ABT solution for 15 min to induce a chemical binding, SERS spectra were measured at 5 different sites of individual fiber substrates. First, the scattered signals directly coming from the fiber-top surface were analyzed to investigate the effect of the distribution of gold nanoparticles on the SERS characteristics. In Figure 4a,b, the highest Raman peak at 1076 cm^−1^ of 4-ABT, which is associated with the a_1_-type vibrational mode [32], was selected as the primary signal. For a concentration of 1 mM, we observed the primary SERS peaks of 7415 ± 570 for a silk fiber and 9337 ± 798 for a multi-mode fiber, respectively, when a laser beam of λ = 785 nm was incident to the fiber-top surfaces directly (black lines). Larger SERS intensity for the multi-mode fiber sample seems to be associated with densely aggregated gold nanoparticles [33]. Contrary to the case of silk-based SERS probes, closely aggregated gold nanoparticles may lead to an enhanced nanogap effect, thereby resulting in a higher density of hotspots on the fiber-top surface. It should also be noted that relatively lower SERS signals for the silk fiber could be improved by optimizing the distribution of gold nanoparticles via liquid-level manipulation in the CSA procedures [25]. SERS enhancement factor (EF) was defined as EF = (*I*_SERS_/*N*_SERS_)/(*I*_BULK_/*N*_BULK_), where *I*_SERS_ and *I*_BULK_ are the SERS and Raman peak intensities at 1076 cm^−1^ for 4-ABT, and *N*_SERS_ and *N*_BULK_ are the numbers of 4-ABT molecules on the SERS substrate and 4-ABT powders within a laser spot volume [34]. Using our experimental conditions, the EF values were 2.31 × 10^4^ for the multi-mode fiber and 1.84 × 10^4^ for the silk one (Supplementary Information). The SERS EF values obtained were in good agreement with previous results by aggregated metallic nanostructures [35].

Second, when the incident laser was coupled into the core/cladding structure of the fiber probes, the SERS intensities at the 4-ABT concentration of 1 mM were 1642 ± 188 and 1794 ± 114 for the silk and multi-mode fibers, respectively, as shown by the red lines in Figure 3d,e. The notable decrease in the SERS signals is attributed to the optical loss after traveling back and forth within the fibers. In addition, it is evident that the proposed silk-based SERS probe can provide a good signal quality, which is comparable to the results of the silica fiber probe.

**Figure 5:**
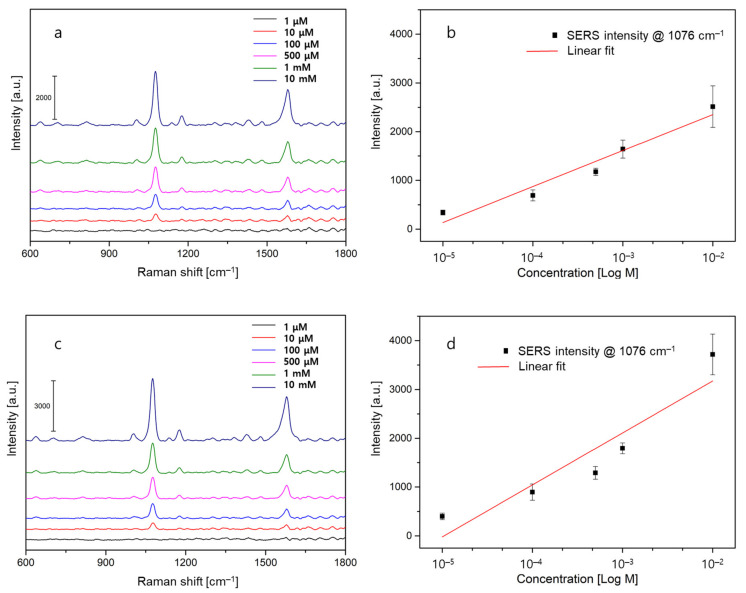
SERS experiments for a varied concentration of 4-ABT molecules. (**a**) SERS measurement data of 4-ABT from 1 µM to 10 mM and (**b**) Linear regression analysis (the red line) of the intensity at 1076 cm^−1^ for silk fiber samples. (**c**) SERS measurement data of 4-ABT and (**d**) Linear regression analysis (the red line) of the intensity at 1076 cm^−1^ for multi-mode fiber samples.

Finally, we explored the sensitivity and linearity by varying the concentration of 4-ABT in the range from 1 µM to 10 mM, as shown in Figure 5. For both fiber-optic SERS probes, we could find the primary SERS peak at a concentration as small as 10 µM in Figure 5a,c indicating no significant difference in the limit of detection. The sensor sensitivity is defined by the ratio of the change of the sensor signal to the change of the actual value of interest. The larger the change of the measured signal for a given change in the actual value, the larger the sensitivity. Here, the sensor sensitivity values can be determined by calculating the slope between the average SERS intensity and the concentration of 4-ABT. From the results in Figure 5b,d sensor sensitivities were found to be 739 and 1065 for the silk and multi-mode fibers, respectively. We speculated that the improved sensitivity of the multi-mode fiber was because of the strong electromagnetic field enhancement by plasmonic nanostructures, particularly in the vicinity of the nanogap regions. Further, in regression, the coefficient of determination (*R*^2^) is a statistical measure of how well the regression predictions approximate the real data points. Based on its formula for linear regression [36], the *R*^2^ value lies between 0 and 1, and the closer it is to 1, the higher the prediction ability. In Figure 5b,d *R*^2^ values were 0.93 for the silk fiber and 0.83 for the multi-mode fiber, respectively. This means that the proposed silk fiber probe can provide a better prediction model with higher linearity and reliability over a wide dynamic range of target concentrations. In short, owing to gold nanoparticles being less densely but more uniformly patterned on top of the silk fiber, the application to linear and reliable SERS detection will be feasible.

## 4. Conclusions

In this study, based on the advantages of biocompatibility and process compatibility of silk fibroin, we successfully developed a novel SERS-active fiber-optic probe by combining a flexible silk fiber with gold nanoparticles. Simple and low-cost CSA technique was employed to realize uniform distribution of gold nanoparticles. High optical transmittance and sensitive SERS detection characteristics of the proposed silk fiber were confirmed experimentally when compared to the results of the silica multi-mode fibers. For the target molecules of 4-ABT, the detection limit was obtained as small as 10 μM, and a fairly good performance in the EF of about 10^4^ and the linearity of 0.93 over a wide dynamic range was achieved. Since the flexible and biocompatible fiber is useful in manipulating and transporting light into the human body, the limitation of in vivo light transmission can be overcome by the development of silk fibroin fibers. It is thus expected that our silk-based SERS probe has the potential for highly sensitive and quantitative detection of disease-related biomolecules.

## Data Availability

The data presented in this study are available on request from the corresponding author.

## Supplementary Materials

The following supporting information can be downloaded at: https://www.mdpi.com/article/10.3390/s22229012/s1, Supplementary Information S1. Detailed explanation of the EF calculation.
