# Supplementary material for: Fabrication and Characterization of Novel Silk Fiber-Optic SERS Sensor with Uniform Assembly of Gold Nanoparticles
Source: Sensors (Basel). 2022 Nov 21;22(22):9012. doi: 10.3390/s22229012 (PMC9692301; doi:10.3390/s22229012)
Supplement: Supplementary file 1 [file sensors-22-09012-s001.zip › sensors-1989278-supplementary.pdf]

Supplementary Information S1: Detailed explanation of the EF calculation.

We describe how to calculate the enhancement factor (EF).

$$\text{EF} = \frac{I_{SERS}/N_{SERS}}{I_{BULK}/N_{BULK}}$$

I_{SERS} = 7415 for the silk fiber and 9337 for the silica fiber

I_{BULK} = 10,095 for the 4-ABT power

$$N_{SERS} = \frac{Total\ surface\ area\ of\ gold\ nanoparticles\ in\ laser\ spot\ size}{Size\ of\ target\ molecule} = 35.55 \times 10^{9}$$

$$N_{BULK} = \frac{D_{molecule} \times Laser\ spot\ size \times Penetration\ depth}{MW_{molecule}} \times 6.022 \times 10^{23} = 89.16 \times 10^{13}$$

Table S1. Calculation parameters

Diameter of laser	100 μm
Penetration depth	20 μm
Density of 4-ABT ($D_{molecule}$)	1.18 g/cm^3
Molecular weight of 4-ABT ($MW_{molecule}$)	125.19 g/mol
Size of 4-ABT	0.2 nm^2
Total surface area of gold nanoparticles	7.11 m^2

$$\text{EF (silk fiber)} = \frac{I_{SERS}}{N_{SERS}} \times \frac{N_{BULK}}{I_{BULK}} = 1.84 \times 10^{4}$$

$$\text{EF (silica fiber)} = \frac{I_{SERS}}{N_{SERS}} \times \frac{N_{BULK}}{I_{BULK}} = 2.31 \times 10^{4}$$
